# Variations in soil phosphorus fractionations in different water-stable aggregates under litter and inorganic fertilizer treatment in Korean pine plantation and its natural forest

**DOI:** 10.1016/j.heliyon.2023.e17261

**Published:** 2023-06-14

**Authors:** Anwaar Hussain, Muhammad Atif Jamil, Kulsoom Abid, Lixin Chen, Kashif Khan, Wenbiao Duan, Tajwar Alam, Umair Riaz

**Affiliations:** a School of Forestry, Northeast Forestry University, Harbin 150040, China; b Key Laboratory of Sustainable Forest Ecosystem Management, Ministry of Education, Northeast Forestry University, Harbin 150040, China; c Department of Natural Resource Management (NRM), National Agricultural Research Center (NARC), Islamabad 44000, Pakistan; d Institute of Soil and Environmental Sciences, Pir Mehr Ali Shah Arid Agriculture University, Rawalpindi 46300, Pakistan; e Department of Soil & Environmental Sciences, MNS University of Agriculture, Multan-60000, Pakistan

**Keywords:** Soil aggregates, Phosphorus availability, Organic P, Inorganic P, PCA analysis

## Abstract

Soil aggregation in forest ecosystem is considered as a significant physical process mainly influenced by manure, fertilizers or combination. This aggregation may directly alter the soil nutrient and their fractions in soil. So, soil samples were collected from two types of forests i.e. Natural Korean pine forests (NKPF) and Korean pine plantation (KPP) in order to know the quantities of organic and inorganic Phosphorus (P) amounts in different aggregate sizes viz. >5 mm, 2–5 mm, 0.25–2 mm, <0.25 mm under forest litter and synthetic fertilizer application below the treatments as undisturbed soil (CK), removed litter (RL), altered litter (AL) while the fertilizer treatments were as control; C: (No added N and P,), L: low (5 g N m^−2^ a^−1^ + 5 g P m^−2^ a^−1^), M: medium (15 g N m^−2^ a^−1^ + 10 g P m^−2^ a^−1^) and H: high concentration (30 g N m^−2^ a^−1^ + 20 g P m^−2^ a^−1^), respectively. The results showed that H_2_O-Pi, NaHCO_3_-Pi, Residual Pi, SOC were highest retained in larger soil aggregates (>5 mm) and decreased with the decreasing aggregate size, while other variables, i.e., NaOH-Pi, NaHCO_3_-Po, pH and T-N were not affected in aggregate size. H_2_O-Pi (48 ppm), NaHCO_3_-Pi (68 ppm), NaHCO_3_-Po (80 ppm), NaOH-Po (623 ppm), HCL-Po (67 ppm), SOC (20.36 ± 1.6) was estimated in medium fertilizer treatment. PCA analysis showed that spread/variance of data points on F1 (62.90%) is more than spread/variance of data points on F2 (57.74%) in NKPF and KPP, respectively, while correlation matrix showed high correlation between H_2_O-Pi and NaOH-Pi (0.63) and H_2_O-Pi and NaHCO_3_-Pi (0.63) while a strong negative correlation was present between Res-Pi and Po (−0.61). Moreover, litter inputs increased the organic-P fractions in soil particularly at medium treatment.

## 1 Introduction

Soil requires extensive and adaptive management practice system for maintaining its health, biodiversity and ecosystem sustenance. In crop production systems and land management among the many others, mineral fertilization is decisive for safeguarding the sufficient nutrient supplies and soil stabilization [1]. Similarly, in forest ecosystem, mineral sustenance in the soil ensures its good productivity capability [2,3]. Literature affirms the progressive role of organic and inorganic fertilization application on soil structure and nutrient bio-availability to the plants [2,4]. Organic manures improve soil physical and chemical properties, soil aggregation, higher water retention capacity and enhances porosity, altogether, soil organic carbon and stimulates microbial activity and releases nutrients [5,6]. The continuous land cropping with no or minimal fertilization can proffer worsen effects on the soil nutrient movement. Studies reveal that P content diminishes in soils when no organic or inorganic fertilizers are added. In this regard, synthetic fertilizers are being used widely as they fulfil plant micro/macro nutrients need, while synthetic fertilizers also lead to organic matter loss in soil [7]. For this purpose, the accumulation of organic manures is inevitably prerequisite for maintaining/improving soil physical and chemical structures [8]. The soils vary in available P in different region as the rock driven mineral phosphorus gets depleted under geological processes [9]. Henceforth, plants require nutritional strategies that targets shifting from mineral based P towards recycling of organic P [10,11]. Under natural conditions, the first limited macronutrient for plant growth is phosphorus [12]. However, plants can combat this problem by P recycling and re-mobilization [13]. Plants can also access P through processes which enhance P uptake efficiencies viz. increase in activities and affinities of P transporters [14,15].

Soil physical structure is likewise an imperative character that defines the soil health and its formation, and amid many factors, soil aggregates is vivacious parameter for assessing the physical structure of soil [16,17]. Essential phases in plant growth and development solely rely on the soil aggregation, in particular, it manages aeration, accessible water and nutrients and their consequent movement [17]. Soil aggregation also regulates soil resistance to erosion which serves as an imperative facet for soil sustainability [18,19]. However, the aggregates formation significantly rely on the existing soil organic matter content and consistent organic modifications makes soil aggregates more stable and recover its physical structure [16]. Literature affirms that soil aggregation progression can critically impact P bio-availability as well as its sorption. Green et al. [20], has stated that manure application, its type, source and management practice direct P forms and distribution in soil. Henceforth, in an effort to boost soil quality, it is critical to genuinely understand the different aggregate sizes and fate of P distribution in them. Wei et al. [8], elaborated that applied fertilizer/manure affects P concentration, distribution and accessibility whereas Curaqueo et al. [21], identified three aspects which influences soil aggregate stability viz. microbial biomass, soil organic carbon and glomalin. They found a strong correlation among these factors and soil aggregate stability. Glomalin is defined as organic substance glycoprotein copiously produced by all arbuscular mycorrhizal fungi, measured operationally in soils as glomalin-related soil protein [22]. Glomalin is firmly incorporated into the hyphae and spore wall in large amounts and is highly positively correlated with soil aggregate stability [23].

In light of these considerations, a study was planned with a focus to evaluate soil P fractionation in two forest types comprising Korean Pine Plantation and its Natural Forest. Our specific aim was to elucidate the impact of forest litter and mineral fertilizations (application of N and P) on P distribution in different soil aggregates. This study is of practical importance as huge areas under forest cover is present and in order to extract maximum benefit through our natural resources, forest litter may be utilized for improving soil health and fertility in a longer run. Specifically, we hypothesized that: (i) forest litter and its quantity have diverse impact on the organic and inorganic phosphorus fractions, soil organic carbon, total nitrogen and soil pH; (ii) forest litter along with synthetic fertilizers can improve the nutrient build-up in the soil, and (iii) diverse soil aggregates have different available P fractions under organic and inorganic amendments.

## 2 Material and methods

### 2.1 Site description

The current investigation was carried out at Liangshui National Reserve, located at the Xiao Xing'an Mountains, Heilongjiang province, North-eastern China. It is situated at 47°6′49″-47°16′10″N, 128°47′8″-128°57′19″E and experiences temperate climate with annual temperature of −0.3 °C. The mean annual precipitation is recorded as 676 mm and most of precipitation (60%) occurs during the months of June–August. The research area comes under the prime areas of China which contains huge natural forests and natural Korean pine forests cover an area of 2375 hm^2^. The experiment comprised of two forest types and their basic characteristics are depicted in Table 1.

**Table 1:** basic characteristics of forest types.

Forest Type	Main species composition	Land use history	Age(year)	Basal area (m^2^/ha)	Mean DBH (cm)	Density (trees/ha)
NKPF	*Pinus koraiensis, Betula costata,* *Tilia amurensis, Acer ukurunduense,* *Abies nephrolepis, Ulmus laciniata,* *Acer tegmentosum*, and *Fraxinus* *Mandshurica*	Natural forest	>300	33.0 (0.9)	11.2 (0.6)	3406 (345)
KPP	*Pinus koraiensis*	Reforestation in 1954 after clear-cutting of primary mixed broad-leaved Korean pine forest	59	41.6 (3.6)	16.0 (1.1)	2072 (116)

### 2.2 Experimental design and treatments

The study site comprised of two forest types viz. Korean pine plantation and its natural Korean pine forest. The overall experiment contained two treatment factors applied in each forest type. There were three main plots on which litter application was made according to the treatments. Litter application was done at three levels i.e., Ck (Undisturbed soil), RL (removed litter) and AL (Alter/double litter). The subplots of undisturbed soil received no litter treatment; the litter from the forest floor was removed in the RL subplots while the removed litter was added into the AL subplot thus making its quantity double. All the main plots were covered with net so, that no further fresh forest litter was added/altered during the experimental duration. Likewise, each main plot was divided into four sub plots that comprised of fertilizer levels as treatments. Each of the four sub plots were treated as control (No added N and P), low (5 g N m^−2^ a^−1^ + 5 g P m^−2^ a^−1^), medium (15 g N m^−2^ a^−1^ + 10 g P m^−2^ a^−1^) and high concentration (30 g N m^−2^ a^−1^ + 20 g P m^−2^ a^−1^). The N source was ammonium sulphate ((NH_4_)_2_SO_4_) whereas both nitrogen and phosphorus source were diammonium phosphate ((NH_4_)_2_HPO_4_). Fertigation was used to apply the fertilizers to the plots according to the prescribed quantities. Fertilizer was applied 5 times in a year.

### 2.3 Soil sampling

The soil sampling was carried out at the end of experiment. Each sample was obtained by selecting three points and then soil sample collection was done from 0 to 20 cm soil depth. All three samples were combined into one composite sample, after that, they were packed into the plastic bags, tagged and immediately transported into the laboratory for further analysis.

**Separation of soil aggregates**: The soil samples were oven dried; all the roots and stones were removed completely by hand and aggregates of different sizes (>5 mm, 2–5 mm, 0.25–2 mm, <0.25 mm) were separated through wet sieving method and then the samples were subjected to oven drying at 105 °C.

### 2.4 Determination of total nitrogen and soil organic carbon

The total nitrogen was determined by Kjeldahl method. For soil organic carbon, the samples were air dried and finely ground, and were passed through 0.149 mm sieve [24]. Two grams of each sample was hydrolysed with 5 mL 1 mol L^−1^ HCl in a 50 mL beaker with repetitive shaking until the soil solution was free from bubbles, it was heated at 60 °C for 2 to 3 h in order to obtain dry weight [24,25]. We subsequently extracted 25 μg of the samples to determine the SOC content using an Element Analyzer (Elementar Vario EL, Hanau, Germany).

### 2.5 Soil phosphorus fractionation

Soil phosphorus fractionation was carried out by applying the method proposed by Sui et al., [26]. The sequential P fractionation was carried out for evaluating the available P fractions which were removed on the basis increasing chemical stability with different labile P fractions. A soil sample of 0.5 g was separated from composite sample of each treatment and sequential extraction was started with deionized water followed by addition of 0.5 M NaHCO_3_ (pH = 8.2), 0.1 M of NaOH and 1 M of HCL. The sample was shaken for 16 h after adding each component. H_2_SO_4_–H_2_O_2_ was used for residual-P digestion. The extracts of NaHCO_3_, NaOH and HCL were divided into two aliquots in order to determine Total phosphorus and inorganic P (Pi). The total determined P was regarded as TP while oxidation with H_2_SO_4_–H_2_O_2_ released Po whereas, Pi was determined prior to digestion with H_2_SO_4_–H_2_O_2_. Po was calculated as the difference between TP and Pi. Moreover, available P (Pa) was extracted by implementing the molybdenum blue method in which 0.03 m NH_4_F and 0.025 M HCL was used. Pi and sodium bicarbonate extracted Po are labile P pools (it also includes some microbial P and they are weakly sorbet on the surfaces of iron, aluminum oxides of P). Labile P included H_2_O–P, NaHCO_3_-Pi, and NaHCO_3_-Po fractions. Pi is the sum of H_2_O-Pi, NaHCO_3_-Pi, NaOH-Pi, HCl-Pi, and Res-P. Po is the sum of NaHCO_3_-Po, NaOH-Po, and HCl-Po.

### 2.6 Statistical analysis

All the collected data was subjected to ANOVA and treatment means were separated by least significant difference at 1.00% probability level (Fisher protected LSD). Principal component analysis (PCA) was further carried out through R software and biplots were generated to compared the correlation among the observed data. The separate correlation analysis was performed on R software.

**Figure:**
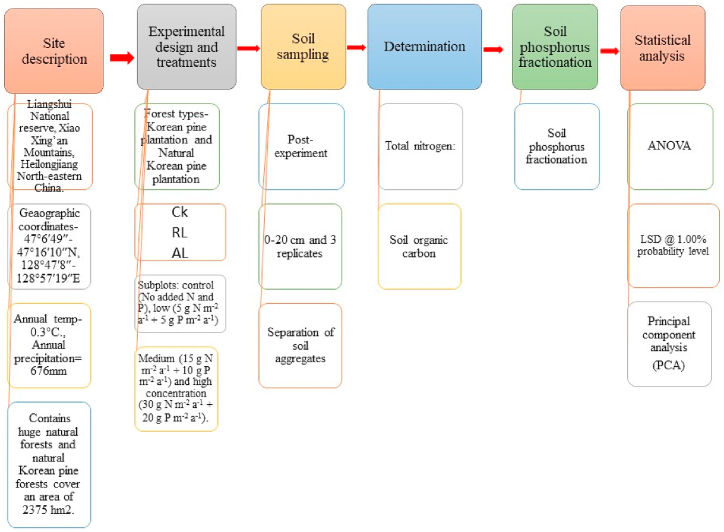

## 3 Results

### 3.1 Outcome of forest litter and mineral fertilization on inorganic P fractions

#### 3.1.1 Effect of forest litter and mineral fertilization on H_2_O Pi

**Fig. 1:**
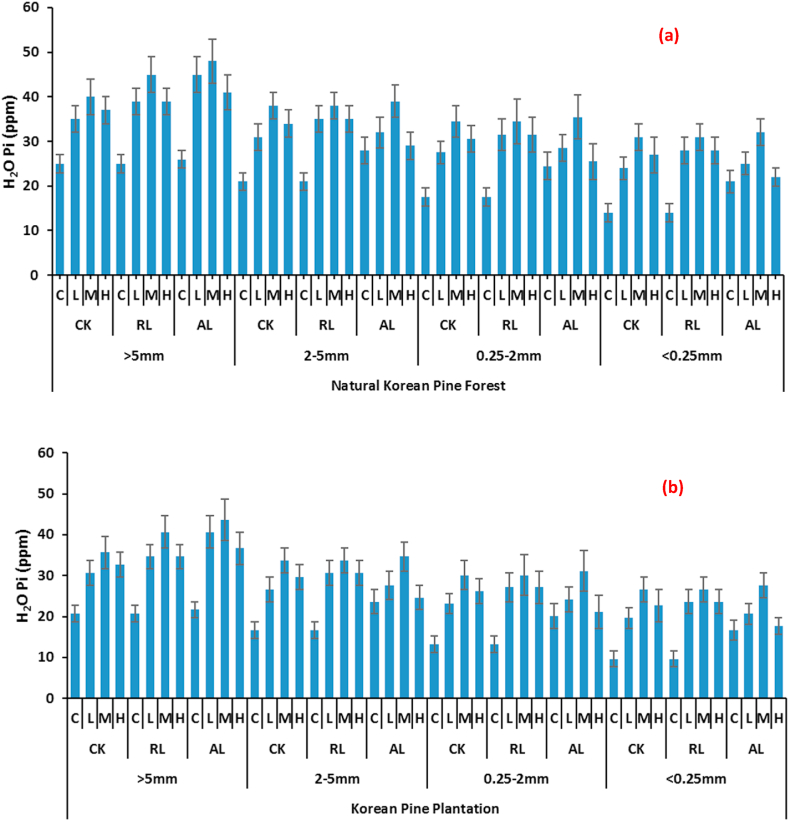
Changes in H_2_O Pi with respect to soil aggregate sizes affected by different litter and N & P treatments at (a) Natural Korean Pine Forest (b) Korean pine Plantation (CK: undisturbed soil, RL: remove litter, AL: alter/double litter, C: No N & P, L: 5 g N m^−2^ a^−1^ + 5 g P m^−2^ a^−1^, M: 15 g N m^−2^ a^−1^ + 10 g P m^−2^ a^−1^, H: 30 g N m^−2^ a^−1^ + 20 g P m^−2^ a^−1^. Values are means ± SE (Tukey's HSD post hoc; P < 0.05).

The results revealed that the distribution of H_2_O-Pi in different soil aggregates differ significantly, additionally all the studied treatment parameters like forest type, litter and fertilizer quantities altered (enhance or decrease) H_2_O-Pi contents. In natural Korean pine forests, the amount of H_2_O-Pi was highest in >5 mm aggregates whereas minimum was noted in <0.25 mm aggregate size. The distribution trend showed that H_2_O-Pi amount was highest in larger aggregates and the quantity decrease with the decrease in aggregate size. The same pattern was observed in other forest type. As far as the effect of forest litter is concerned, it showed that forest litter can give highest H_2_O-Pi quantities. The highest value was observed in AL: alter/double litter while no litter was less effective in enhancing the H_2_O-Pi quantities. Moreover, inorganic fertilizers also affected H_2_O-Pi distribution; the highest value (48 ppm) was obtained in Medium (M: 15 g N m^−2^ a^−1^ + 10 g P m^−2^ a^−1^) treatment while the least (26 ppm) was obtained in control (CK: undisturbed soil) in AL treated plots. In artificial Korean pine plantation, the distribution pattern was same, however, the highest value was 43.65 (>5 mm, altered, medium treatment) while under control it was 21.65 ppm. The detailed result of all treatments is depicted in Fig. 1 (a, b).

#### 3.1.2 Effect of forest litter and mineral fertilization on NaHCO_3_-Pi

**Fig. 2:**
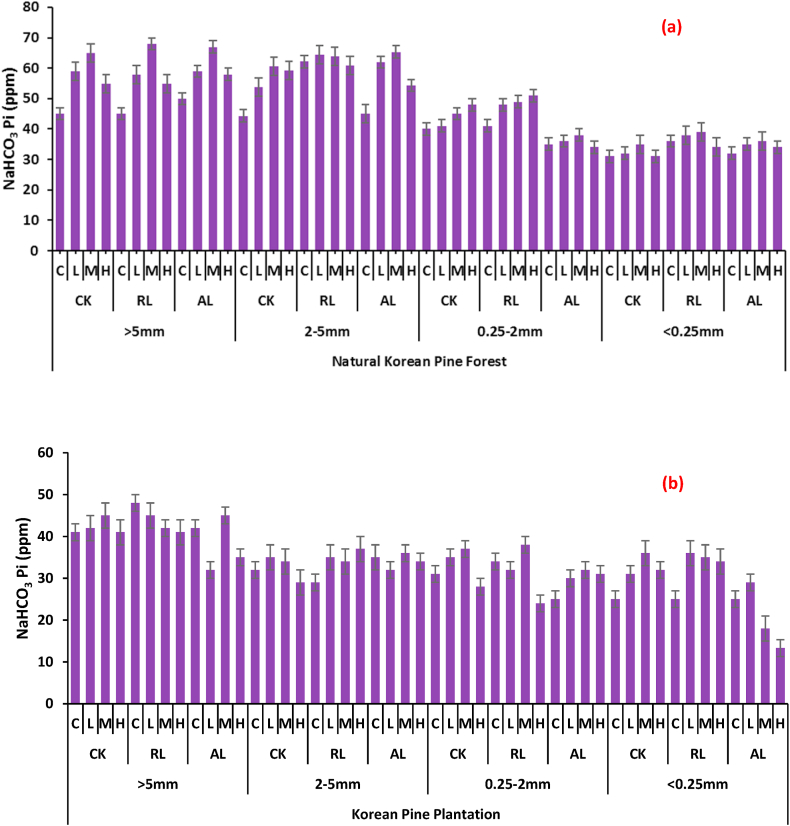
Changes in NaHCO_3_ Pi with respect to soil aggregate sizes affected by different litter and N & P treatments at (a) Natural Korean Pine Forest (b) Korean Pine Plantation (*CK: undisturbed soil, RL: remove litter, AL: alter/double litter, C: No N & P, L:* 5 g N m^−2^*a*^*−1*^*+ 5 g P m*^*−2*^*a*^*−1*^*, M:* 15 g N m^−2^*a*^*−1*^*+ 10 g P m*^*−2*^*a*^*−1*^*, H:* 30 g N m^−2^*a*^*−1*^*+ 20 g P m*^*−2*^*a*^*−1*^*. Values are means ± SE (Tukey's HSD post hoc; P < 0.05).*

Our study showed that higher quantities of NaHCO_3_-Pi existed in macro aggregates (>5 mm and 2–5 mm) and decreased in aggregates (0.25–2 mm, <0.25 mm) in natural Korean pine forest. In macro aggregates (>5 mm) the highest quantity (68 ppm) was present in CK (M: 15 g N m^−2^ a^−1^ + 10 g P m^−2^ a^−1^) followed by AL under medium fertilizer (67 ppm) while the minimum was noted in control plots, however the quantity decreased with the decrease in aggregate size, the least value of 31 ppm was noted in treatment where no litter as well as no fertilizer was applied. As far as artificial Korean pine plantation, is concerned, our result showed a much lesser available quantity of *NaHCO*_*3*_*-Pi* than that of natural one. For instance the highest value was 48 ppm in aggregates size >5 mm (forest litter, no fertilizer application) followed by 45 ppm (alter litter, medium fertilizer). The detailed results are depicted in Fig. 2 (a, b).

#### 3.1.3 Effect of forest litter and mineral fertilization on NaOH-Pi

**Fig. 3:**
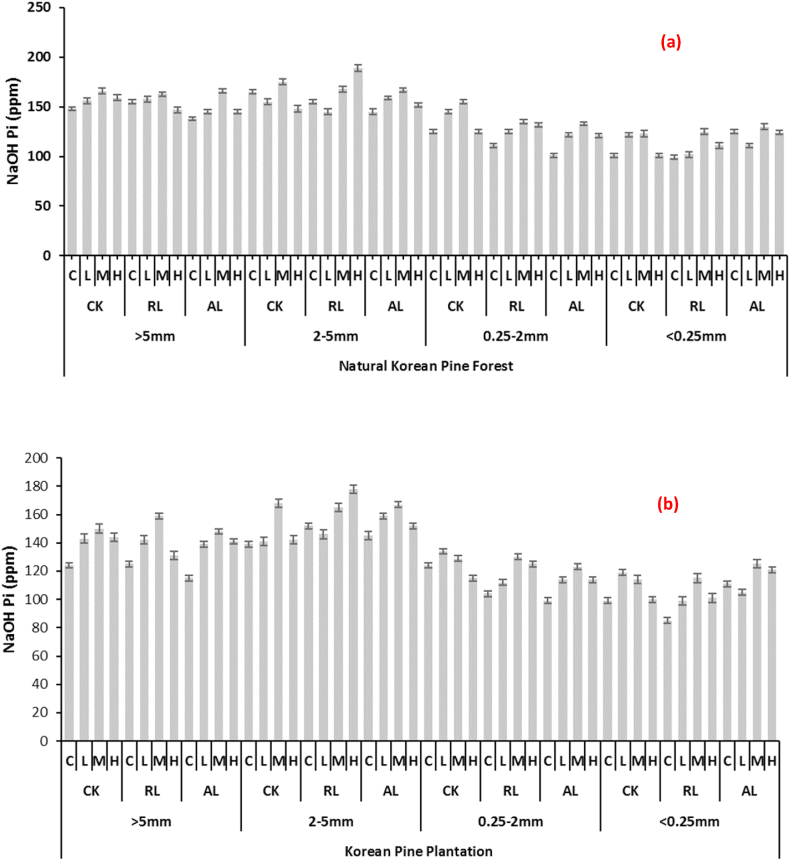
Changes in NaOH Pi with respect to soil aggregate sizes affected by different litter and N & P treatments at (a) Natural Korean Pine Forest (b) Korean Pine Plantation (CK: undisturbed soil, RL: remove litter, AL: alter/double litter, C: No N & P, L: 5 g N m^−2^ a^−1^ + 5 g P m^−2^ a^−1^, M: 15 g N m^−2^ a^−1^ + 10 g P m^−2^ a^−1^, H: 30 g N m^−2^ a^−1^ + 20 g P m^−2^ a^−1^) Values are means ± SE (Tukey's HSD post hoc; P < 0.05).

In Natural Korean pine plantation, the amount of NaOH-Pi varied among the soil particle size as well as the differently treated fertilizer plots. The highest amounts were obtained in 2–5 mm aggregates followed by > 5 mm whereas in 0.25–2 mm and <2 mm aggregates the quantities decreased. As far as the litter treatments are concerned, there was no significant difference among the mean values, however the fertilizer treatment (H*:* 30 g N m^−2^ a^−1^ + 20 g P m^−2^ a^−1^) gave highest value of 189 ppm. The similar distribution trend was observed in Korean pine plantation; however, the values differ in each treatment. The detailed results are shown in Fig. 3 (a, b).

#### 3.1.4 Effect of forest litter and mineral fertilization on residual-Pi

**Fig. 4:**
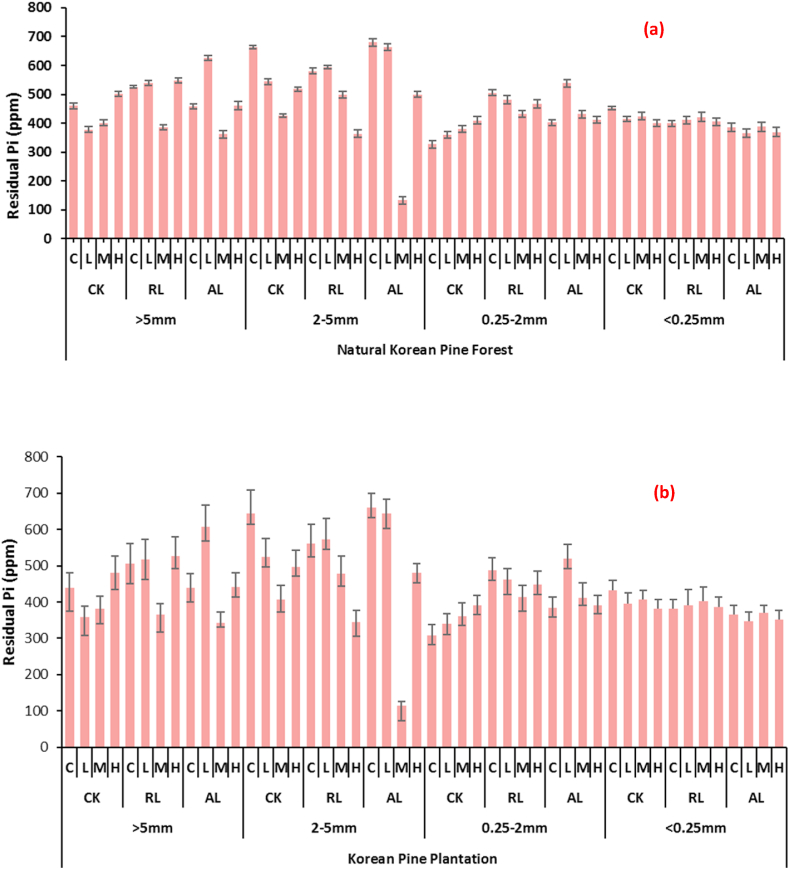
Changes in Residual Pi with respect to soil aggregate sizes affected by different litter and N & P treatments at (a) Natural Korean Pine Forest (b) Korean Pine Plantation (CK: undisturbed soil, RL: remove litter, AL: alter/double litter, C: No N & P, L: 5 g N m^−2^ a^−1^ + 5 g P m^−2^ a^−1^, M: 15 g N m^−2^ a^−1^ + 10 g P m^−2^ a^−1^, H: 30 g N m^−2^ a^−1^ + 20 g P m^−2^ a^−1^) Values are means ± SE (Tukey's HSD post hoc; P < 0.05).

Residual Pi was found to be in high amounts in larger soil aggregates i.e., >5 mm and 2–5 mm whereas a general decrease was observed in 0.25–2 mm and <0.25 mm aggregates. As far as the litter application is concerned then no significant difference was observed in different treatments. The similar pattern was observed in artificial Korean pine plantations. In natural Korean pine forest, the highest residual Pi (680 ppm) was found in 2–5 mm aggregate size where litter was double in which no N&P was applied. In Korean pine plantation the highest was also observed in same treatment while the value was 661 ppm. The overall result of each treatment is presented in Fig. 4 (a, b).

### 3.2 Effect of litter and mineral fertilizers on organic P fractions

**Fig. 5:**
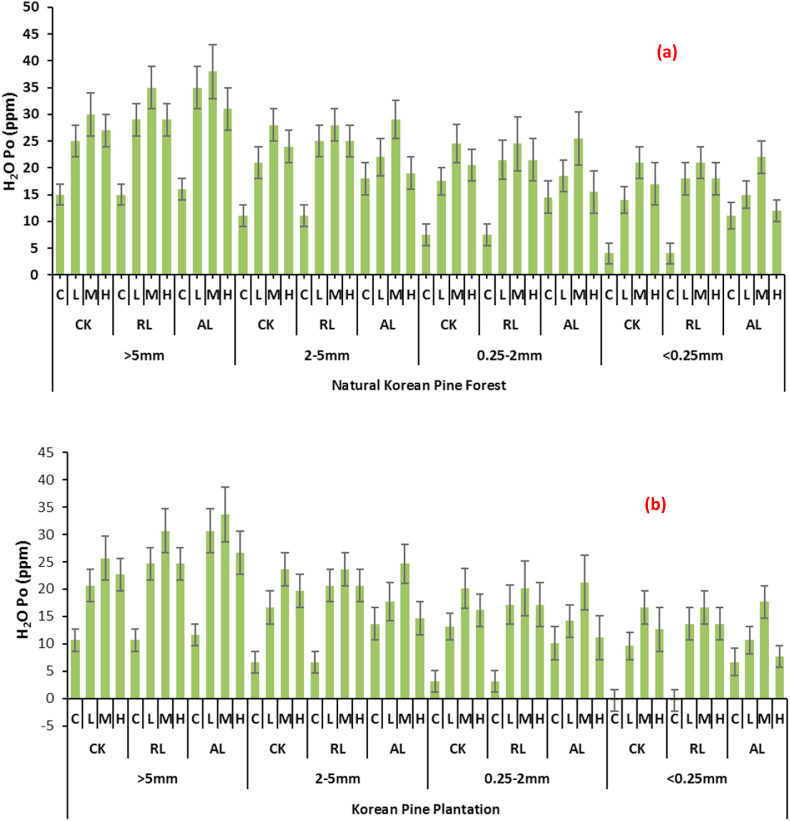
Changes in H_2_O–Po Residual Pi with respect to soil aggregate sizes affected by different litter and N & P treatments at (a) Natural Korean Pine Forest (b) Korean Pine Plantation (CK: undisturbed soil, RL: remove litter, AL: alter/double litter, C: No N & P, L: 5 g N m^−2^ a^−1^ + 5 g P m^−2^ a^−1^, M: 15 g N m^−2^ a^−1^ + 10 g P m^−2^ a^−1^, H: 30 g N m^−2^ a^−1^ + 20 g P m^−2^ a^−1^) Values are means ± SE (Tukey's HSD post hoc; P < 0.05).

Organic P fractions were affected under the influence of litter as well as N and P however their presence did not differ significantly with regard to aggregate size. In natural Korean pine plantation, the NaHCO_3_-Po was affected only by the mineral application. The highest quantities were observed under the medium applied fertilizer, while the least were observed in control. Whereas low and high application gave less values as compared to medium application. The highest value of 80 ppm was observed medium treatment whereas a least value was 50 in case of control. The similar trend was observed in Korean pine plantation. The detailed results are depicted in Table 2 (a, b). As far as NaOH-Po is concerned it was also retained highest in treatments where N and P were applied under medium treated plots while litter application did not affect the quantities significantly. Moreover, the presence in different aggregates also did not vary significantly Table 2 (a, b). H_2_O–Po was found more amounts in larger soil aggregates i.e., >5 mm and 2–5 mm whereas a general decrease was observed in 0.25–2 mm and <0.25 mm aggregates. As far as the litter application is concerned then no significant difference was observed in different treatments. The similar pattern was observed in artificial Korean pine plantations. It was noted that overall, the H_2_O–Po values were greater in natural Korean pine forest as compared to Korean pine plantation the highest was also observed in same treatment while the value was 661 ppm. The result of each treatment is depicted in Fig. 5 (a, b).

**Table 2 (a):** Changes in different organic P fractions, pH, SOC and T-N with respect to soil aggregate sizes affected by different litter and N & P treatments at Natural Korean Pine Forest.

Forest Type	size	Litter	N & P	NaHCO_3_-P_o_	NaOH-P_o_	HCl-P_o_	pH	SOC	T-N
	(mm)			(ppm)				(g kg^−1^)	
Natural Korean Pine Forest	>5	CK	C	50 ± 3.2	405 ± 20.2	23 ± 1.25	5.76 ± 0.92	13.21 ± 1.02	1.02 ± 0.01
			L	60 ± 4.1	523 ± 30.2	26 ± 1.1	5.65 ± 0.99	17.23 ± 1.03	1.05 ± 0.01
			M	70 ± 5.3	550 ± 32.3	34 ± 1.6	5.64 ± 0.99	18.26 ± 1.01	1.09 ± 0.02
			H	60 ± 4.2	460 ± 26.5	30 ± 1.5	5.61 ± 1.02	11.23 ± 0.99	0.98 ± 0.01
		RL	C	65 ± 4.5	442 ± 40.7	35 ± 1.3	5.68 ± 0.85	13.02 ± 0.94	1.03 ± 0.02
			L	71 ± 6.3	467 ± 36.2	18 ± 0.7	5.65 ± 0.51	18.12 ± 0.99	1.08 ± 0.04
			M	75 ± 7.4	547 ± 46.9	38 ± 1.3	5.61 ± 0.45	19.23 ± 0.98	1.11 ± 0.03
			H	64 ± 5.3	476 ± 31.4	19 ± 0.9	5.62 ± 0.63	15.41 ± 1.01	0.99 ± 0.01
		AL	C	70 ± 7.0	530 ± 39.5	28 ± 1.6	5.67 ± 0.38	15.36 ± 1.06	1.04 ± 0.02
			L	75 ± 4.3	486 ± 46.5	18 ± 0.6	5.64 ± 0.48	19.34 ± 1.05	1.10 ± 0.03
			M	78 ± 5.8	573 ± 47.2	26 ± 1.3	5.61 ± 0.63	21.56 ± 1.00	1.19 ± 0.04
			H	74 ± 4.9	506 ± 60.0	25 ± 1.4	5.00 ± 0.47	16.02 ± 1.05	1.05 ± 0.03
	>2-5	CK	C	74 ± 6.0	387 ± 21.5	30 ± 1.6	5.71 ± 0.36	11.95 ± 0.78	1.03 ± 0.02
			L	72 ± 5.4	452 ± 41.8	17 ± 0.6	5.68 ± 0.25	15.97 ± 0.96	1.04 ± 0.04
			M	76 ± 4.6	527 ± 50.4	31 ± 1.3	5.65 ± 0.45	17.00 ± 0.78	1.05 ± 0.03
			H	72 ± 5.3	499 ± 47.6	23 ± 1.3	5.61 ± 0.36	9.97 ± 0.69	0.99 ± 0.02
		RL	C	60 ± 6.1	411 ± 40.4	29 ± 1.2	5.69 ± 0.12	11.74 ± 0.41	1.05 ± 0.04
			L	75 ± 4.6	438 ± 43.1	18 ± 0.8	5.67 ± 0.36	16.74 ± 0.63	1.06 ± 0.05
			M	80 ± 5.9	522 ± 48.1	30 ± 1.8	5.66 ± 0.14	17.74 ± 0.41	1.07 ± 0.06
			H	76 ± 4.1	480 ± 40.5	20 ± 1.1	5.62 ± 0.36	13.74 ± 0.25	1.01 ± 0.05
		AL	C	60 ± 6.1	388 ± 32.5	18 ± 0.9	5.72 ± 0.55	13.74 ± 0.36	1.12 ± 0.05
			L	71 ± 5.8	328 ± 34.5	29 ± 2.1	5.66 ± 0.89	17.74 ± 0.55	1.06 ± 0.06
			M	76 ± 6.4	582 ± 40.6	37 ± 2.7	5.64 ± 0.78	19.74 ± 0.58	1.04 ± 0.04
			H	72 ± 6.7	519 ± 50.2	27 ± 1.9	5.63 ± 0.68	14.74 ± 0.63	1.00 ± 0.06
	>0.25–2.0	CK	C	58 ± 4.6	500 ± 47.6	24 ± 1.6	5.36 ± 0.74	12.59 ± 0.47	0.97 ± 0.05
			L	62 ± 6.1	521 ± 41.2	25 ± 2.1	5.33 ± 0.47	16.61 ± 0.63	1.01 ± 0.07
			M	63 ± 5.6	535 ± 43.5	26 ± 2.3	5.30 ± 0.68	17.64 ± 0.45	1.02 ± 0.06
			H	59 ± 4.6	523 ± 47.5	24 ± 2.1	5.26 ± 0.36	10.61 ± 0.85	0.99 ± 0.08
		RL	C	52 ± 4.6	500 ± 41.6	28 ± 1.6	5.34 ± 0.48	12.38 ± 0.36	0.98 ± 0.02
			L	59 ± 5.3	525 ± 45.6	29 ± 2.6	5.32 ± 0.76	17.38 ± 0.41	1.05 ± 0.06
			M	62 ± 6.1	535 ± 40.2	35 ± 2.1	5.31 ± 0.85	18.38 ± 0.25	1.06 ± 0.06
			H	59 ± 4.9	511 ± 42.3	29 ± 1.6	5.27 ± 0.69	14.38 ± 0.63	1.05 ± 0.04
		AL	C	61 ± 4.3	599 ± 41.2	31 ± 1.5	5.37 ± 0.48	14.38 ± 0.45	0.90 ± 0.03
			L	65 ± 6.2	589 ± 55.6	31 ± 1.9	5.31 ± 0.69	18.38 ± 0.63	1.05 ± 0.05
			M	67 ± 6.2	547 ± 57.9	39 ± 2.1	5.29 ± 0.71	20.38 ± 0.41	1.09 ± 0.01
			H	69 ± 6.3	585 ± 51.6	31 ± 1.6	5.28 ± 0.39	15.38 ± 0.25	1.11 ± 0.03
	<0.25	CK	C	55 ± 5.1	626 ± 57.8	55 ± 2.5	5.67 ± 0.41	13.6 ± 0.39	1.04 ± 0.01
			L	59 ± 4.6	600 ± 59.6	35 ± 1.5	5.66 ± 0.69	17.62 ± 0.41	1.23 ± 0.02
			M	61 ± 6.3	632 ± 46.4	15 ± 0.9	5.59 ± 0.37	18.65 ± 0.55	1.10 ± 0.03
			H	68 ± 6.8	625 ± 46.8	46 ± 1.1	5.62 ± 0.41	11.62 ± 0.62	1.08 ± 0.04
		RL	C	61 ± 3.8	650 ± 56.4	18 ± 0.9	5.74 ± 0.56	13.39 ± 1.02	0.99 ± 0.01
			L	65 ± 4.5	600 ± 49.3	44 ± 4.0	5.71 ± 0.45	18.39 ± 0.99	1.02 ± 0.01
			M	67 ± 3.8	600 ± 55.9	41 ± 3.7	5.69 ± 0.36	19.39 ± 0.78	1.05 ± 0.02
			H	62 ± 3.4	499 ± 41.6	45 ± 3.6	5.64 ± 0.48	15.39 ± 0.66	1.00 ± 0.01
		AL	C	68 ± 4.1	601 ± 60.0	56 ± 4.2	5.71 ± 0.46	15.39 ± 0.78	0.95 ± 0.01
			L	69 ± 3.8	588 ± 48.9	63 ± 3.6	5.63 ± 0.48	19.39 ± 0.76	0.96 ± 0.01
			M	78 ± 3.9	300 ± 24.5	67 ± 3.4	5.65 ± 0.43	21.39 ± 0.48	0.97 ± 0.01
			H	71 ± 4.9	600 ± 51.6	48 ± 2.3	5.63 ± 0.50	16.39 ± 0.90	0.99 ± 0.01

**Table 2 (b):** Changes in different organic P fractions, pH, SOC and T-N with respect to soil aggregate sizes affected by different litter and N & P treatments at Korean Pine Plantation.

Forest	size	Litter	N & P	NaHCO_3_-P_o_	NaOH-P_o_	HCl-P_o_	pH	SOC	T-N
	(mm)			(ppm)				(g kg^−1^)	
Korean Pine Plantation	>5	CK	C	50 ± 2.1	405 ± 30	23 ± 0.9	5.6 ± 0.9	12.21 ± 1.2	1.00 ± 0.01
			L	60 ± 2.3	523 ± 51	26 ± 1.1	5.49 ± 0.8	16.23 ± 1.0	1.03 ± 0.01
			M	70 ± 3.5	550 ± 48	34 ± 1.2	5.48 ± 0.6	17.26 ± 1.3	1.07 ± 0.03
			H	60 ± 2.6	460 ± 48	30 ± 1.3	5.45 ± 0.7	10.23 ± 1.1	0.96 ± 0.01
		RL	C	65 ± 2.1	442 ± 45	35 ± 1.2	5.52 ± 0.5	12.01 ± 1.2	1.01 ± 0.02
			L	71 ± 3.6	467 ± 30	18 ± 1.0	5.49 ± 0.9	17.00 ± 1.2	1.06 ± 0.01
			M	75 ± 3.1	547 ± 46	38 ± 0.9	5.45 ± 0.7	18.21 ± 1.0	1.09 ± 0.03
			H	64 ± 2.5	476 ± 36	19 ± 1.2	5.46 ± 0.6	14.36 ± 1.2	0.97 ± 0.01
		AL	C	70 ± 3.9	530 ± 44	28 ± 1.1	5.51 ± 08	14.55 ± 1.1	1.02 ± 0.02
			L	75 ± 3.1	486 ± 49	18 ± 0.7	5.48 ± 0.9	18.25 ± 0.9	1.08 ± 0.01
			M	78 ± 3.5	573 ± 43	26 ± 0.6	5.45 ± 0.4	20.36 ± 1.6	1.17 ± 0.06
			H	74 ± 1.3	506 ± 19	25 ± 0.8	4.84 ± 0.4	15.14 ± 0.8	1.03 ± 0.01
	>2-5	CK	C	74 ± 2.1	387 ± 24	30 ± 0.6	5.55 ± 0.5	10.95 ± 0.3	1.01 ± 0.01
			L	72 ± 3.6	452 ± 42	17 ± 1.0	5.52 ± 0.6	14.97 ± 0.4	1.02 ± 0.06
			M	76 ± 3.2	527 ± 19	31 ± 0.9	5.49 ± 0.4	16.89 ± 0.6	1.03 ± 0.03
			H	72 ± 3.6	499 ± 46	23 ± 0.5	5.45 ± 0.8	8.97 ± 0.6	0.97 ± 0.01
		RL	C	60 ± 2.4	411 ± 43	29 ± 0.6	5.53 ± 0.6	10.74 ± 1.0	1.03 ± 0.05
			L	75 ± 3.2	438 ± 28	18 ± 0.4	5.51 ± 0.8	15.74 ± 0.9	1.04 ± 0.06
			M	80 ± 4.3	522 ± 29	30 ± 0.8	5.5 ± 0.9	16.74 ± 0.4	1.05 ± 0.02
			H	76 ± 3.6	480 ± 36	20 ± 0.6	5.46 ± 0.7	12.74 ± 0.6	0.99 ± 0.01
		AL	C	60 ± 3.1	388 ± 22	18 ± 1.0	5.56 ± 0.5	12.74 ± 0.8	1.10 ± 0.03
			L	71 ± 2.6	328 ± 21	29 ± 0.8	5.5 ± 0.9	16.74 ± 0.6	1.04 ± 0.04
			M	76 ± 3.1	582 ± 41	37 ± 1.2	5.48 ± 0.4	18.74 ± 0.7	1.02 ± 0.02
			H	72 ± 3.5	519 ± 26	27 ± 1.3	5.47 ± 0.9	13.74 ± 0.8	0.98 ± 0.06
	>0.25–2.0	CK	C	58 ± 3.1	500 ± 26	24 ± 1.2	5.2 ± 0.4	11.59 ± 0.6	0.95 ± 0.04
			L	62 ± 3.6	521 ± 22	25 ± 1.3	5.17 ± 0.2	15.61 ± 0.9	0.99 ± 0.02
			M	63 ± 3.8	535 ± 27	26 ± 1.5	5.14 ± 0.6	16.64 ± 0.4	1.01 ± 0.03
			H	59 ± 3.1	523 ± 23	24 ± 1.4	5.1 ± 0.6	9.61 ± 0.6	0.97 ± 0.02
		RL	C	52 ± 3.6	500 ± 21	28 ± 1.3	5.18 ± 0.4	11.38 ± 0.8	0.96 ± 0.01
			L	59 ± 1.6	525 ± 28	29 ± 1.5	5.16 ± 0.5	16.38 ± 0.9	1.03 ± 0.02
			M	62 ± 1.6	535 ± 54	35 ± 1.3	5.15 ± 0.6	17.38 ± 0.7	1.04 ± 0.02
			H	59 ± 3.2	511 ± 29	29 ± 1.2	5.11 ± 0.5	13.38 ± 0.6	1.03 ± 0.01
		AL	C	61 ± 2.6	599 ± 23	31 ± 1.5	5.21 ± 0.7	13.38 ± 0.5	0.88 ± 0.02
			L	65 ± 2.9	589 ± 44	31 ± 1.6	5.15 ± 0.6	17.38 ± 0.8	1.03 ± 0.01
			M	67 ± 3.0	547 ± 46	39 ± 1.2	5.13 ± 0.8	19.38 ± 0.7	1.07 ± 0.02
			H	69 ± 3.1	585 ± 36	31 ± 1.5	5.12 ± 0.4	14.38 ± 0.6	1.09 ± 0.01
	<0.25	CK	C	55 ± 2.8	626 ± 51	55 ± 1.9	5.51 ± 0.5	12.6 ± 0.9	1.02 ± 0.02
			L	59 ± 2.6	600 ± 46	35 ± 1.7	5.5 ± 0.9	16.62 ± 1.2	1.21 ± 0.02
			M	61 ± 2.7	632 ± 52	15 ± 0.9	5.43 ± 0.8	17.65 ± 1.3	1.08 ± 0.02
			H	68 ± 2.8	625 ± 48	46 ± 1.4	5.46 ± 07	10.62 ± 0.5	1.06 ± 0.02
		RL	C	61 ± 2.6	650 ± 43	18 ± 1.0	5.58 ± 0.6	12.34 ± 0.7	0.97 ± 0.01
			L	65 ± 2.4	600 ± 41	44 ± 1.6	5.55 ± 0.5	17.41 ± 1.3	1.00 ± 0.01
			M	67 ± 2.6	600 ± 49	41 ± 1.3	5.53 ± 0.4	18.39 ± 1.2	1.03 ± 0.01
			H	62 ± 2.8	499 ± 55	45 ± 1.2	5.48 ± 0.6	14.56 ± 1.3	0.98 ± 0.01
		AL	C	68 ± 2.8	601 ± 41	56 ± 1.5	5.55 ± 0.5	14.39 ± 1.0	0.93 ± 0.01
			L	69 ± 2.6	588 ± 43	63 ± 1.6	5.47 ± 0.4	18.39 ± 1.2	0.94 ± 0.01
			M	78 ± 2.4	300 ± 46	67 ± 1.4	5.49 ± 0.5	20.39 ± 1.3	0.95 ± 0.01
			H	71 ± 2.9	600 ± 42	48 ± 1.2	5.47 ± 0.6	15.39 ± 1.2	0.97 ± 0.01

Values are means ± SE (Tukey's HSD post hoc; P < 0.05).

### 3.3 Effect of forest litter and mineral fertilization on SOC, TN and pH

In natural Korean pine plantation, the pH of the soil in different aggregates had a significant change under the influence of application of inorganic fertilizers. In all treatments, a general decrease in pH values was observed with increase in the fertilizer rates. For instance, the value was found to be 5.76 ± 0.92 in control plots where no litter was altered (>5 mm) which decreased to a value of 5.61 ± 1.02 in High application level of N and P, moreover the litter application in all aggregate sizes did not change the pH values up to significant levels. The similar trend was observed in Korean pine plantations; however, the overall pH values suggested that soils were slightly acidic in nature in comparison to natural Korean pine plantations. The detailed results are shown in Table 2 (a, b). Fertilizer treatments affected SOC to a greater extend as the control plots have lesser quantities of SOC which became higher with increases the doses, however SOC was highest up to medium treatment and again decreases at higher doses. A linear increase was observed when we investigated the effect of litter. It was highest in plots where the litter was doubled. The values were obtained as >5 mm (21.56 ± 1.00), >2–5 mm (19.74 ± 0.58), 0.25–2.0 mm (20.38 ± 0.41) and <0.25 mm (21.39 ± 0.48) in natural Korean pine forest. T-N also showed the same trend as that of SOC i.e., under addition of litter highest value of T-N was noted and under medium applied fertilizer the peak values were obtained in all aggregates. The detailed results are shown in Table 2 (a, b).

### 3.4 PCA and correlation analysis

Principal component analysis was performed and two principal components (axis F1 and F2) were identified which represent the highest percentage of variability. In both forest types we selected PC1 and PC2 as they represented 62.90% and 57.74% of the total variations in NKPF and KPP forests respectively. The first biplot is called correlation circle which showed that how the initial variables are projected into factor space. The interpretation was made on the basis of vector distance from the central point and the angle between them. The vectors which were far from the centre and closer to each other represented a positive correlation while orthogonal angles showed no correlation (in our results no variables were at right angles with each other) while the parameters with larger angle and opposite to each other revealed a significant negative correlation. For example, in NKPF forest, NaHCO_3_-Pi and NaOH-Pi were positively correlated whereas as Res-Pi and Po have large angles between them hence they were negatively correlated with each other. Similarly, NaHCO_3_-Po and H_2_O-Pi were close to each other revealing a positive correlation whereas in KPP forest, HCL-Pi was correlated to NaHCO_3_-Po, NaHCO_3_-Po was correlated to H_2_O-Pi.

**Fig. 6:**
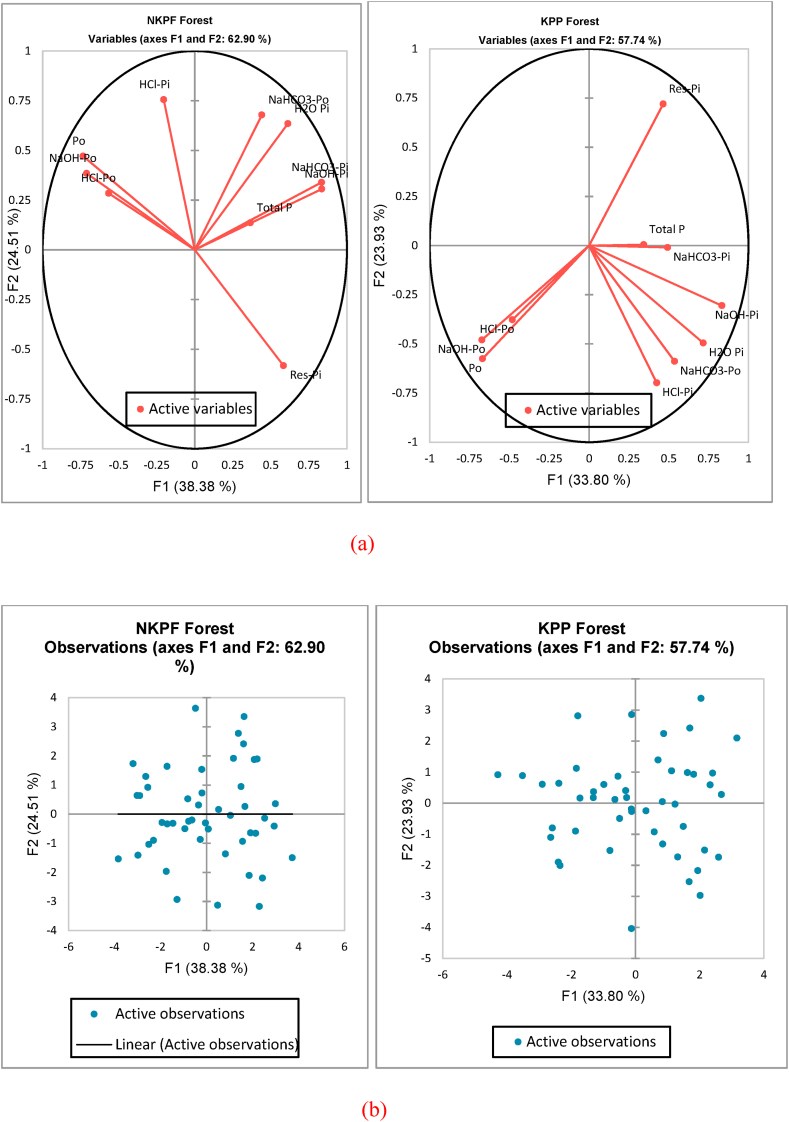
(a) Principal component analysis biplot showing the distribution of data points between the active variables (axes F1 and F2). (b) Principal component analysis biplot showing the distribution of data points between the active observations (axes F1 and F2).

**Fig. 7:**
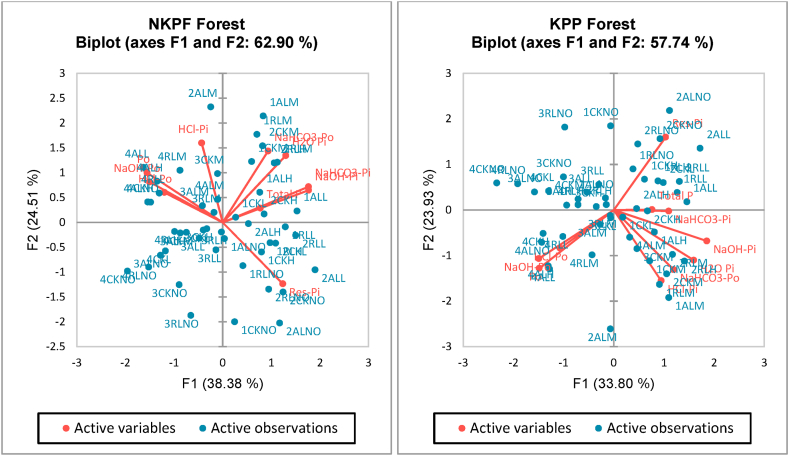
(a, b) Principal component analysis biplot showing the distribution of data points between the active observations and variables (axes F1 and F2).

**Fig. 8:**
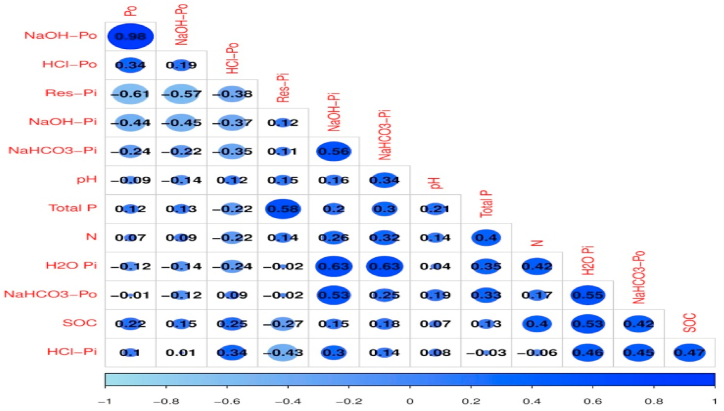
Correlation matrix among soil P fractions.

In case of KPP forest similar trend was found (negative correlation between Po and Res-Pi). In this way all the observed values were linked with each other on the basis of vector length and vector angles Fig. 5 (a, b). The next biplot was generated which show the uniqueness of the treatments and their combinations. It showed that treatments which are in the same quadrant and near to each other have similar effects on the measured variable. However, the points which were away to the other ones showed that they carry uniqueness in their results Fig. 6 (a, b). Fig. 7(a and b) show combination of variables and observations. The correlation matrix was generated and results of correlation coefficients between different P types in soil aggregates. The lower scale numbering showed the correlation coefficients of P types. The results showed that coefficient values which were >0.24 indicated a strong correlation with 95% confidence level. The shades represent the weight of correlation. Dark blue shades represent higher coefficients and decrease with the decrease in shade. The matrix showed that a positive high correlation was present between H_2_O-Pi and NaOH-Pi (0.63) and H_2_O-Pi and NaHCO_3_-Pi (0.63) while a strong negative correlation was present between Res-Pi and Po (−0.61). All other parameters and their coefficients are depicted in Fig. 8 (a, b).

## 4 Discussion

### 4.1 Impact of litterfall on organic P fractions

Organic P as the major form of P is mineralized by the extracellular phosphate enzymes (produced by plant roots and microorganisms) as well as microbial activities. Po recycling is important as plant roots can assess this form easily for their growth and development. As in our results, the organic P was increased under the double litter suggesting that more substrate available for microorganism thus enhancing their activities. It is also suggested that in order to increase P cycling in forests, the release of inorganic phosphate through decomposition of organic phosphorus is necessary that can occur from forest floor or soil organic matter [27].

Our studies are in line with those of Lee et al. [28], and Hu et al. [29], as they showed a significant increase in organic P and labile contents under the application of synthetic fertilizer application (NPK) The reason for varied nutrient quantities (nitrogen, phosphorus, carbon etc.) in aggregates is because aggregates of different sizes form microenvironments for their respective microbial communities [30,31] and represent microbial communities at micro level [32,33]. Naveed et al. [34], stated that soil aggregate fractionation has the ability for potentially and direct diffusion of gases like oxygen and carbon dioxide under the influence of mineral fertilization, which further affects the functions as well as structure of microbial communities associated with aggregates [[35], [36], [37]]. Hence, soil aggregation, mineral availability and organic content are responsible for varied nutrient distribution in the soil, which further leads to differences in gas diffusion among the soil aggregates. These factors ultimately leads to differences in abundance and activities of P related bacteria. The circulation of the P fractions is primarily controlled by the redox potential of soil, soil reaction (pH), dissolved oxygen (DO), organic matter content (OM), microbial community population, and both Fe–Al-oxides and OH^−^ [38,39].

### 4.2 Impact of litterfall on inorganic P fractions

The high levels of extractable P in soils are likely to be more susceptible for losses via runoff whereas higher amounts of extractable P in soil particles have more release potential [40]. Research carried out revealed that labile P (bicarbonate extractable) was found to be in high amount in micro aggregates of sizes 0.125–0.25 mm, 0.053–0.125 mm and 0.053 under the influence of organic amendment an account for 11 to 22% of total inorganic P present in these aggregates [41], however, the presence of labile P in micro aggregates can pose environmental concerns as they have higher ability to release in water via wind or soil erosion. Previously Zhang et al. [42] estimated the higher amount of water-P and bicarbonate P in micro aggregates and also suggested that release from such particle size is expected to be faster. There exists a relationship between particle size and bioactive P amounts as a study by Green et al. [20] elaborated that higher bioactive P was present in macro aggregates whereas the smaller aggregates contained less bioactive P forms which is also in line with our findings. While Yan et al. [43], stated that swine manure (organic fertilizer) had the ability to enhance non-apatite inorganic phosphorus amounts. Apart from the P availability, long term fertilization influences fractionation in soil aggregates by forming mineral organic complexes and higher amounts of arbuscular mycorrhizal fungi [37,[44], [45], [46], [47]].

The phosphorus in inorganic form is mainly bond to secondary minerals and sometimes taken by plant roots or microorganisms e.g. Ref. [48], hence owing to this fact the concentrations of inorganic P are generally less in soil solution, studies have revealed that soils with organic amendments can give lower amounts of inorganic P [49,50]. In another study it was revealed that inorganic P amounts were not decreased in soil with the passing years, however at the end of experiment (after 6 years) the lower Pi concentrations tends to occur in litter [51].

### 4.3 Impact of litterfall on soil nutrient availability

There is literature that affirms the positivity of organic amendments in soil and improved soil aggregation as a result [8,52]. Organic amendments alone as well as in combination with NPK nutrition enhanced the organic carbon contents in aggregates. However, there are cases which states that NPK fertilizer had no effect on OC in aggregates in Chinese red soil. The studies are in accordance with our results that highest amount of organic carbon was observed in macro aggregates i.e., >5 mm. Ahmed et al. [7], stated that OC in ferrosols (23.7 g/kg) and isohumosols (50.2 g/kg) existed in macro aggregates. Six et al. [53], revealed that macro aggregates comprised of micro aggregates as well as organic binding agents due to which the OC increased in macro aggregates as compared to micro aggregates. Others studies are also present that shows a strong correlation between SOC and aggregates stability, as aggregates are more cohesive due to organic polymer binding agents [54,55]. However, there are also studies which revealed that organic fertilizers may not influence micro aggregates and remained similar to unfertilised (control) [56,57]. The opposite results on aggregate stability may be due to the specific soil characteristics and climatic conditions (Yu et al., 2012).

## 5 Conclusions

In forests, finding feasible and most suitable methods for enhancing soil physiochemical properties is very necessary. Among many, application of organic matter in either form is a promising soil management practice that improves soil structure, improve microbial population and activities. In return microbe actions helps in release of nutrients which otherwise are bounded to soil particles. This bioavailability of nutrients increases the health of ecosystem positively. In addition, the application of synthetic fertilizers in optimum quantities can augment the availability of different form of Phosphorus in soil. Conclusively our research findings makes forest litter as a promising source for enhancing soil physical and biochemical properties. As we focused on the availability of organic and inorganic phosphorus fractions, Altered/double litter application to the soil was most beneficial for soil health. Contrastingly, the absence of forest litter does not improved the availability of phosphorus in either form. However, it is also important to use inorganic fertilisers as microbial communities requires some energy to start their processes for mineralization of organic contents. Similarly, soil aggregates of different sizes have different capacities for storing mineral nutrients. By using the results from this study, further management processes can help the forest managers to efficiently incorporate the otherwise non useable forms of forest litter in their bioavailable form.

## Author contribution statement

Anwaar Hussain, Muhammad Atif Jamil and Kulsoom Abid: Contributed reagents, materials; Performed the experiments and Wrote the paper.

Lixin Chen & Wenbiao Duan: Conceived and designed the experiments.

Kashif Khan, Tajwar Alam and Umair Riaz: Analyzed and interpreted the data; Contributed analysis tools.

## Data availability statement

Data will be made available on request.

## Additional information

No additional information is available for this paper.

## Declaration of competing interest

The authors declare that they have no known competing financial interests or personal relationships that could have appeared to influence the work reported in this paper.
